# A Prognostic Nomogram Based on Immune Scores Predicts Postoperative Survival for Patients with Hepatocellular Carcinoma

**DOI:** 10.1155/2020/1542394

**Published:** 2020-07-07

**Authors:** Jukun Wang, Chao Zhang, Ang Li, Feng Cao, Dongbin Liu, Fei Li, Tao Luo

**Affiliations:** Department of General Surgery, Xuanwu Hospital of Capital Medical University, No. 45 Changchun Street, Xicheng District, Beijing 100053, China

## Abstract

**Background:**

Increasing research attention has focused on tumor-infiltrating immune cells. However, the threshold of an immune score for use in predicting overall survival (OS) and disease-free survival (DFS) in hepatocellular carcinoma (HCC) is not defined. This study aims at exploring the association between immune scores with prognosis and building a clinical nomogram for predicting the survival of HCC patients. *Material and Methods*. A total of 299 patients were enrolled in this study. Their clinical pathological characteristics and immune scores downloaded from The Cancer Genome Atlas (TCGA) database were analyzed. Survival differences between different immune score subgroups were compared, and a final nomogram was built using the Cox proportional hazards regression model. The predictive performance of the nomogram was assessed using the concordance index (C-index) and a calibration plot.

**Results:**

All the patients were divided into three subgroups based on immune scores. Patients with medium and high immune scores had significantly better OS (HR and 95% CI: 0.417 [0.186-0.937] and 0.299 [0.146-0.616]) and DFS (HR and 95% CI: 0.575 [0.329-1.004] and 0.451 [0.278-0.733], respectively, compared with those with low immune scores. The C indices for OS and DFS were 0.748 (95% CI, 0.687-0.809) and 0.675 (95% CI, 0.630-0.720), respectively. A calibration plot used to determine the probability of survival at 3 or 5 years (OS and DFS) showed a significant agreement between nomogram predictions and actual observations.

**Conclusions:**

Medium and high immune scores are significantly associated with prolonged OS and DFS in HCC patients. Nomograms built in this study can help doctors and patients assess prognosis and guide treatment.

## 1. Background

Hepatocellular carcinoma (HCC) is one of the most common malignant tumors. Among the major cancers, it is ranked the sixth highest with regard to incidence and the third highest in terms of mortality [1]. China has a high incidence of primary liver cancer, ranking the fourth in terms of malignant tumor incidence and the second in the mortality rate. Infection by chronic hepatitis B virus represents the primary risk factor [2, 3]. A lack of obvious clinical symptoms during the early stage, coupled with the occult occurrence, means that most patients diagnosed in the middle to late stages miss the opportunity for surgery. Although surgery represents the most effective treatment, a high rate of metastasis and postoperative recurrence remains an obstacle for the long-term survival of HCC patients.

HCC prognosis depends on tumor factors, liver function, and general condition of the patient. Conventional prognostic assessments of HCC patients are based on the tumor-node-metastasis (TNM) staging system, which considers tumor extent, lymph node invasion, and detectable metastasis. Although the TNM staging system is essential for treatment and prognosis, it provides limited information in the prediction of the postoperative outcomes of HCC patients. Moreover, many patients with the same tumor stages have significantly different clinical outcomes [4]. In recent years, increasing evidence has indicated that the host immune system is significantly correlated to cancer development and influences clinical prognosis [5, 6].

Several studies have been conducted to explore the relationship between tumor microenvironment and patient prognosis. Malignant solid tumor tissues consist not only of tumor cells but also tumor-associated normal epithelial, immune, stromal, and vascular cells. Immune and stromal cells have been shown to be important for tumor growth, invasion, and metastasis in ovarian, pancreatic, and colorectal cancers, as well as gastric adenocarcinoma [7–11]. Furthermore, Yoshihara et al. [12] calculated immune and stromal scores using gene expression signatures and used them to infer immune and stromal cell fractions in tumor samples. Although these scores have great potential of predicting prognosis and guiding treatment strategies, the specific immune cells and intricate mechanisms that affect the tumor development are not elucidated and therefore require further research.

To our knowledge, reports describing the relationship between immune scores and HCC prognosis are nonexistent. Therefore, this study aimed at evaluating the association between immune scores with prognosis and building a clinical nomogram for predicting the survival of HCC patients.

## 2. Materials and Methods

### 2.1. Materials

In this study, all data were downloaded from The Cancer Genome Atlas (TCGA) database, which is currently the largest database available for genomic analyses of tumors. The dataset includes clinical information on at least 20 types of cancer [13]. Clinical pathological information from the TCGA was downloaded from an open-access resource that included a unique number of patient, sex, age, Edmondson-Steiner grade, TNM stage, overall survival (OS) status, OS time, disease-free survival (DFS) status, and DFS time [14].

### 2.2. Data Preprocessing

In cases where replicate and incomplete data were identified, we excluded all records from further analyses. Each immune score corresponded to one patient. In total, 299 patients were included in the analysis. The data of immune scores associated with HCC patients were downloaded from the ESTIMATE (Estimation of STromal and Immune cells in MAlignant Tumor tissues using Expression data) database directly.

### 2.3. Statistical Analysis

We used OS and DFS as the primary endpoints. Overall survival was defined as the interval from diagnosis to death from any cause, while DFS was defined as the interval from the time of surgery to initial tumor relapse or death. The cut off value for immune score was decided using the X-tile software (Yale University School of Medicine, New Haven, CT, USA) [15]. Specifically, we visualized the best cut-point and predicted population subsets (low, medium, and high immune score subgroups) based on the relationship between the immune score and overall survival status by an X-tile plot. A chi-square test was used to calculate differences between immune score subgroups and other clinical pathological variables. The Kaplan-Meier method was used to construct survival curves, while differences between immune score subgroups were tested using the log-rank test. This was done to explore the difference between immune score subgroups and prognosis, with all analyses performed using packages implemented in R software 3.6.1. Univariate and multivariate Cox proportional hazards regression models were used to calculate hazard ratios for OS and DFS, with only variables that attained a *p* < 0.05 threshold, in the univariate analysis, entered into the multivariate analysis.

Nomograms were constructed based on clinical pathological variables, then subjected to 1000 bootstrap resamples for interval validation of the analyzed database. The concordance index (C-index) and calibration plot were used to evaluate the performance of the nomogram during prognosis prediction [16].

All tests were two-sided, and *p* < 0.05 was considered statistically significant. All statistical analyses were performed using R 3.6.1 and SPSS 22.0 software.

## 3. Results

### 3.1. Patient Characteristics

A total of 299 patients (210 men, and 89 women) were included in this study. We observed no association between clinical pathological variables and immune scores (Table 1). The average age of patients was 58.36 years (SD = 13.16, range 16-84), with grade stage distribution as follows: grade 1, 40 (13.38%), grade 2, 145 (48.49%), grade 3, 104 (34.78%), and grade 4, 10 (3.34%). TNM stage distribution of patients used in this study was as follows: stage I 151 (50.50%), stage II 72 (24.08%), stage III 72 (24.08%), and stage IV 4 (1.34%).

The cut-off points for the immune scores were -786.8 and -402.9, respectively. Consequently, patients were subsequently subdivided into low, medium, and high immune score subgroups. In total, 30 (10.03%) patients recorded scores lower than or equal to -786.8 (low immune score subgroup), 61 (20.4%) had scores between -786.8 and -402.9 (medium immune score subgroup), while 208 (69.57%) had scores greater than -402.9 (high immune score subgroup). We found median OS and DFS times of 20.4 and 13.63 months, respectively. In addition, the average ages of patients across different immune score subgroups were 58.32, 58.21, and 58.36 years, respectively.

### 3.2. Univariate and Multivariate Analyses of OS and DFS

**Figure 1:**
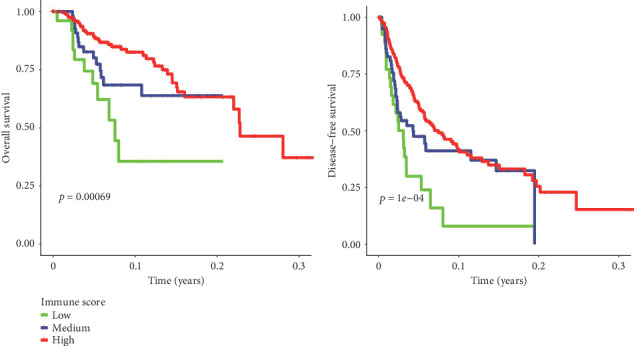
Kaplan-Meier curves describing the association of immune scores with OS (left) and DFS (right) for patients with hepatocellular carcinoma. Red line represents high immune score subgroup (>-402.9). Blue line represents medium immune score subgroup (-786.8 to -402.9). Green line represents low immune score subgroup (≤-786.8). The *p* = 0.00069 at OS and *p* = 1*e* − 04 at DFS are presented, respectively.

Results from the univariate Cox regression analysis were shown in Table 2. We found statistical significance among TNM stages and immune scores for OS and DFS. In addition, age was significantly associated with DFS, but sex and grade were not associated with clinical prognosis. There were significant differences in OS and DFS between patients with low, medium, and high immune scores (*p* < 0.05) (Figure 1).

We performed the Cox multivariate regression analysis using age, TNM stage, and immune score (Table 3). The TNM stage and immune scores remained significantly associated with OS and DFS, while age was associated only with DFS. Patients with medium and high immune scores had significantly better OS (HR and 95% CI: 0.417 [0.186-0.937] and 0.299 [0.146-0.616]) and DFS (HR and 95% CI: 0.575 [0.329-1.004] and 0.451 [0.278-0.733], respectively, compared to those with low immune scores. Moreover, when compared with those with TNM stage I, patients with stage II, stage III, and stage IV had worse OS (HR and 95% CI: 1.332 [0.641-2.768], 4.218 [2.397-7.421], and 13.552 [2.939-62.485], respectively) and DFS (HR and 95% CI: 2.078 [1.396-3.095], 2.717 [1.854-3.981], and 7.634 [2.246-25.951]), respectively. Finally, compared with patients who were less than 30 years old, those who were 41-50 and 61-70 years of age were associated with better DFS (HR and 95% CI: 0.397 [0.170-0.930] and 0.456 [0.218-0.952]), respectively.

### 3.3. Prognostic Nomogram for OS and DFS

**Figure 2:**
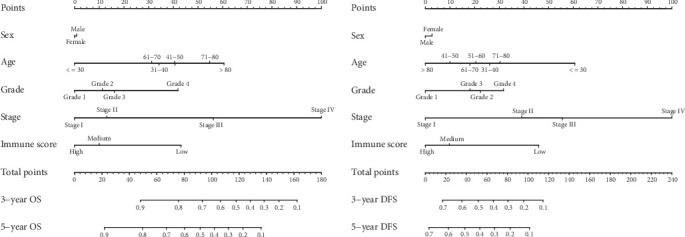
Hepatocellular carcinoma OS (left) and DFS (right) nomograms at 3- and 5-year that integrated clinical pathological characteristics and immune score. The point axis represents a value corresponding to a score of every factor including sex, age, grade, stage, and immune score. The total point axis represents a value corresponding to total score of all factors. Survival axis represents the likelihood of 3- and 5-year survival according to total score.

**Figure 3:**
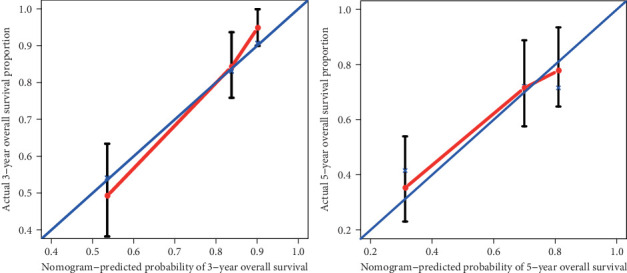
OS calibration curve at 3- (left) and 5-year (right) for patients with hepatocellular carcinoma. *x*-axis represents nomogram-predicted probability of 3- and 5-year OS. *y*-axis represents actual 3- and 5-year OS.

**Figure 4:**
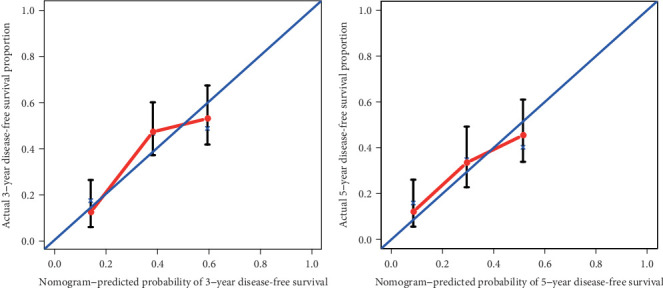
DFS calibration curve at 3- (left) and 5-year (right) for patients with hepatocellular carcinoma. *x*-axis represents nomogram-predicted probability of 3- and 5-year DFS. *y*-axis represents actual 3- and 5-year DFS.

The prognostic nomogram, that integrated all clinical pathological factors for OS and DFS, was shown in Figure 2. The C-indices for OS and DFS were 0.748 (95% CI, 0.687-0.809) and 0.675 (95% CI, 0.630-0.720), respectively. For the agreement evaluation between the nomogram prediction and actual observation, the calibration plots visualized the results, respectively (Figures 3 and 4). For the probability of OS at 3 or 5 years, the calibration plot presented a good agreement between prediction and actual observation. For the probability of DFS at 3 or 5 years, although the calibration plot also described a good agreement generally, there was a slight deviation compared with the OS.

## 4. Discussion

In this study, we evaluated the prognostic value of immune score in patients with HCC. Results from univariate and multivariate analyses of OS and DFS revealed that both medium and high immune scores were significantly associated with good prognosis. In addition, we integrated all clinical pathological factors in the construction of a clinical nomogram and predicted the survival of patients with HCC.

The TNM staging system is a traditional classification tool, based on the tumor invasion parameter, that effectively estimates the prognosis of patients with a variety of cancers [17]. However, this system has some limitations related to prognostic information. First, its classification only focuses on invasive tumor process and fails to incorporate the potential effect of a patient's immune system [18]. Secondly, the prognostic outcome can significantly vary among patients with similar histological tumor stages [4].

Accumulating data from human cancers have demonstrated that immune classification could serve to evaluate prognosis and guide development of treatment options, owing to advances in microarray-based gene expression profiling technology coupled with an in-depth understanding of tumor-infiltrating immune cells. For instance, Galon et al. [10] investigated the relationship between the type, density, and location of immune cells within tumors and the clinical outcome of colorectal cancer patients and reported that adaptive immune cell infiltration had a superior prognostic value to, and independent of, classical migration and invasion tumor criteria. They further analyzed these immune cells in relation to tumor evolution and clinical outcome and proposed an “immunoscore” for quantifying the density of CD3+ and CD8+ T cells in the tumor center as well as its invasive margin. This has become a key platform for immune classification in colorectal cancer patients [19, 20]. Similarly, the immune score has been recently shown to be a strong prognostic factor and an indicator of chemosensitivity in patients with advanced serous ovarian cancer [8].

In addition to the prognostic value of immune cell infiltration, the role of predicting therapeutic responses has also been established. Denkert et al. [21], while investigating the relationship between lymphocyte infiltration in breast cancer and the response to neoadjuvant chemotherapy, reported that the presence of tumor-associated lymphocytes was an independent predictor of response to anthracycline/taxane chemotherapy. Similarly, Halama et al. [22] analyzed the localization and density of immune cells in the invasive margin of colorectal cancer and liver metastases. They found tumor-infiltrating lymphocytes could predict response to chemotherapy in metastatic colorectal cancer. Numerous studies also provided evidence for the role of tumor-infiltrating lymphocytes in predicting therapeutic response [8, 23, 24].

Tumor-infiltrating immune cells are highly correlated with prognosis and immunotherapy in patients with HCC. Furthermore, the treatment response to anticancer therapies depends on the degree and distribution of tumor-infiltrating immune cells. Recently, Rohr-Udilova et al. [25] have assessed the relative proportions of immune cells in healthy human livers, HCC samples, and adjacent tissues by deconvoluting gene expression microarray data. They reported a higher fraction of total T, B, and naïve B cells in both HCC and HCC-adjacent tissues than in healthy liver tissue. They further identified T follicular helper and memory B cells as the involved T and B cell subsets. To gain a better understanding into the complex relationships between T and B cells within HCC tissues, Garnelo et al. [26] demonstrated that the depletion of B cells resulted in enhanced tumor growth and reduced local T cell activation, and that the interaction between T and B cells played an important role in activating their function and controlling tumor growth. In China, Li et al. [27] constructed an immune type system based on the signature of immune cells infiltration in HCC and reported this system could predict overall survival and disease-free survival for patients with HCC effectively. Similarly, Hu et al. [28] proposed a novel systemic immune-inflammation index (SII) based on lymphocyte, neutrophil, and platelet counts to predict the prognosis of HCC patients. They found this index can be used as an independent factor for prognosis, and it even showed higher prediction ability compared with other conventional pathological characteristics. In our study, we also confirmed that the immune score can be used as an independent prognostic factor for HCC patients. Furthermore, we found that high immune scores were correlated with prolonged OS and DFS in HCC patients. This could be attributed to the fact that high immune scores represent more immune cell infiltration, which activates an enhanced immune system and play a role as an antitumor [14, 25].

To the best of our knowledge, this is the first nomogram predicting OS and DFS in patients with HCC based on immune scores and clinical pathological characteristics. Moreover, the nomogram can play an important role in, easily and individually, the prognosis of patients at different risk stages, and this might decide treatment options. However, there are potential shortcomings to our study. First, we did not perform an external validation due to the lack of data for use in calculating immune scores. Secondly, we integrated a low number of personal characteristics which could affect the accuracy of this clinical prediction model. Finally, due to the lack of complete treatment information for HCC patients in the TCGA, we were unable to adjust for the effect of treatment on prognosis.

## 5. Conclusions

Our findings demonstrated that medium and high immune scores were significantly related to prolonged OS and DFS in HCC patients. In addition, we constructed and validated a nomogram for use in predicting prognosis for HCC patients at different risk stages. In clinical practice, this nomogram could help doctors to effectively advise patients on their survival and treatment options.

## Figures and Tables

**Table 1:** Association between clinical pathological characteristics and immune scores in 299 hepatocellular carcinoma patients.

Characteristics		Immune score				
	Total	Low (≤-786.8)	Medium (-786.8 to -402.9)	High (>-402.9)	*X* ^2^	*p*
Sample sizes	299	30 (10.03)	61 (20.40)	208 (69.57)	—	—
Sex					1.302	0.522
Female	89	11 (36.67)	20 (32.79)	58 (27.88)		
Male	210	19 (63.33)	41 (6.56)	150 (72.12)		
Age (years)					17.233	0.141
≤30	13	3 (10.00)	3 (4.92)	7 (3.37)		
31-40	16	1 (3.33)	4 (6.56)	11 (5.29)		
41-50	36	3 (10.00)	9 (14.75)	24 (11.54)		
51-60	88	11 (36.67)	16 (26.23)	61 (29.33)		
61-70	99	8 (26.67)	19 (31.15)	72 (34.62)		
71-80	44	2 (6.67)	10 (16.39)	32 (15.38)		
>80	3	2 (6.67)	0 (0.00)	1 (0.48)		
Grade					8.502	0.204
1	40	6 (20.00)	5 (8.20)	29 (13.94)		
2	145	8 (26.67)	33 (54.10)	104 (50.00)		
3	104	15 (50.00)	20 (32.79)	69 (33.17)		
4	10	1 (3.33)	3 (4.92)	6 (2.88)		
Stage					11.544	0.073
I	151	10 (33.33)	25 (40.98)	116 (55.77)		
II	72	8 (26.67)	16 (26.23)	48 (23.08)		
III	72	11 (36.67)	20 (32.79)	41 (19.71)		
IV	4	1 (3.33)	0 (0.00)	3 (1.44)		

**Table 2:** Univariate analyses of the OS and DFS of hepatocellular carcinoma patients according to clinical pathological characteristics and immune scores.

		OS				DFS			
Characteristics	Total	Alive	Dead	HR (95% CI)	*p*	Disease-free	Recurred	HR (95% CI)	*p*
Sex									
Female	89	65 (28.02)	24 (35.82)	1.000		37 (26.81)	52 (32.30)	1.000	
Male	210	167 (71.98)	43 (64.18)	0.824 (0.498, 1.364)	0.452	101 (73.19)	109 (67.70)	0.828 (0.595, 1.152)	0.262
Age (years)									
≤30	13	12 (5.17)	1 (1.49)	1.000		4 (2.90)	9 (5.59)	1.000	
31-40	16	12 (5.17)	4 (5.97)	1.222 (0.135, 11.070)	0.859	6 (4.35)	10 (6.21)	0.317 (0.127, 0.790)	0.014
41-50	36	28 (12.07)	8 (11.94)	1.594 (0.198, 12.810)	0.661	20 (14.50)	16 (9.94)	0.284 (0.125, 0.650)	0.003
51-60	88	69 (29.74)	19 (28.36)	1.747 (0.233, 13.080)	0.587	42 (30.43)	46 (28.57)	0.373 (0.182, 0.766)	0.007
61-70	99	80 (34.48)	19 (28.36)	1.438 (0.192, 10.780)	0.724	47 (34.06)	52 (32.30)	0.336 (0.164, 0.686)	0.003
71-80	44	29 (12.50)	15 (22.39)	2.146 (0.282, 16.340)	0.461	18 (13.04)	26 (16.15)	0.376 (0.175, 0.807)	0.012
>80	3	2 (0.86)	1 (1.49)	3.153 (0.197, 50.560)	0.417	1 (0.72)	2 (1.24)	0.418 (0.090, 1.948)	0.267
Grade		232	67						
1	40	32 (13.79)	8 (11.94)	1.000		19 (13.77)	21 (13.04)	1.000	
2	145	115 (49.57)	30 (44.78)	1.159 (0.531, 2.533)	0.711	70 (50.72)	75 (46.58)	1.197 (0.733, 1.954)	0.471
3	104	78 (33.62)	26 (38.81)	1.348 (0.609, 2.984)	0.461	44 (31.88)	60 (37.27)	1.267 (0.769, 2.086)	0.353
4	10	7 (3.02)	3 (4.48)	2.035 (0.534, 7.750)	0.298	5 (3.62)	5 (3.11)	1.253 (0.470, 3.341)	0.652
Stage									
I	151	130 (56.03)	21 (31.34)	1.000		88 (63.77)	63 (39.13)	1.000	
II	72	60 (25.86)	12 (17.91)	1.503 (0.783, 3.059)	0.261	29 (21.01)	43 (26.71)	2.171 (1.468, 3.209)	<0.001
III	72	40 (17.24)	32 (47.76)	4.379 (2.517, 7.617)	<0.001	20 (14.49)	52 (32.30)	2.928 (2.019, 4.246)	<0.001
IV	4	2 (0.86)	2 (2.99)	8.774 (2.038, 37.779)	0.004	1 (0.72)	3 (1.86)	8.832 (2.726, 28.620)	**<**0.001
Immune score									
Low	30	19 (8.19)	11 (16.42)	1.000		8 (5.80)	22 (13.66)	1.000	
Medium	61	46 (18.83)	15 (22.39)	0.456 (0.208, 0.997)	0.049	27 (19.57)	34 (21.12)	0.538 (0.314, 0.923)	0.024
High	208	167 (71.98)	41 (61.19)	0.289 (0.147, 0.570)	<0.001	103 (74.64)	105 (65.22)	0.381 (0.239, 0.606)	<0.001

**Table 3:** Multivariate analyses of the OS and DFS of hepatocellular carcinoma patients according to clinical characteristics and immune scores.

Characteristics	OS		DFS	
	HR (95% CI)	*p*	HR (95% CI)	*p*
Age (years)				
≤30	1.000		1.000	
31-40	2.733 (0.299, 25.008)	0.373	0.549 (0.215, 1.403)	0.210
41-50	3.054 (0.373, 25.009)	0.298	0.397 (0.170, 0.930)	0.033
51-60	3.338 (0.440, 25.321)	0.244	0.507 (0.240, 1.067)	0.074
61-70	2.427 (0.317, 18.607)	0.394	0.456 (0.218, 0.952)	0.037
71-80	4.465 (0.577, 34.578)	0.152	0.582 (0.262, 1.291)	0.183
>80	5.027 (0.290, 87.030)	0.267	0.393 (0.080, 1.927)	0.249
Stage				
I	1.000		1.000	
II	1.332 (0.641, 2.768)	0.443	2.078 (1.396, 3.095)	<0.001
III	4.218 (2.397, 7.421)	<0.001	2.717 (1.854, 3.981)	<0.001
IV	13.552 (2.939, 62.485)	0.001	7.634 (2.246, 25.951)	0.001
Immune score				
Low	1.000		1.000	
Medium	0.417 (0.186, 0.937)	0.034	0.575 (0.329, 1.004)	0.052
High	0.299 (0.146, 0.616)	0.001	0.451 (0.278, 0.733)	0.001

## Data Availability

All clinical pathological data of HCC patients can be downloaded directly from the cBioPortal database at: http://www.cbioportal.org/. All immune score data of HCC patients can be downloaded directly from the ESTIMATE database at: https://bioinformatics.mdanderson.org/estimate/. In addition, all codes used for data analysis are available from the corresponding author on responsible request.
